# Left Ventricular Strains and Myocardial Work in Adolescents With Anorexia Nervosa

**DOI:** 10.3389/fcvm.2022.798774

**Published:** 2022-02-08

**Authors:** Justine Paysal, Etienne Merlin, Daniel Terral, Aurélie Chalard, Emmanuelle Rochette, Philippe Obert, Stéphane Nottin

**Affiliations:** ^1^Avignon University, LAPEC EA4278, Avignon, France; ^2^CHU Clermont-Ferrand, Néonatologie et Réanimation Pédiatrique, Clermont-Ferrand, France; ^3^CHU Clermont-Ferrand, Pédiatrie, Clermont-Ferrand, France; ^4^Université Clermont Auvergne, INSERM, CIC 1405, Unité CRECHE, Clermont-Ferrand, France; ^5^CHU Clermont-Ferrand, Cardiologie, Clermont-Ferrand, France

**Keywords:** myocardial strain, myocardial work, left ventricular mechanical dispersion, anorexia nervosa, left ventricular twist

## Abstract

**Background:**

Anorexia nervosa (AN) is accompanied by bradycardia, low blood pressure (BP) and cardiac morphological remodeling. Systolic and diastolic functions are relatively preserved when assessed by standard ultrasound methods. However, novel advances based on speckle tracking echocardiography (STE), that could detect subtle and early alterations of left ventricular (LV) function, remained poorly used in AN patients.

**Objective:**

The aim of this study was to assess the cardiac function of AN patients by evaluating LV myocardial strains, myocardial work (MW) and LV mechanical dispersion. We hypothesized that LV strains and global myocardial work would be decreased and LV twisting mechanisms enhanced to preserve the systolic function.

**Methods:**

Fifty-nine adolescents including 26 women AN patients (14.6 ± 1.9 yrs. old) with a mean duration of AN of 19 ± 9 months and 33 controls (14.1 ± 2.0 yrs. old) underwent STE to assess LV morphology and myocardial regional strains.

**Results:**

The global longitudinal strain (GLS) was higher in AN patients compared to controls (−18.8 ± 2.0 vs. −16.9 ± 2.8%, *p* = 0.006). The area under the pressure-strain loop, representing the global MW was not altered but was shifted to the left and downwards in AN patients, due to their lower BP and higher GLS. Intraventricular mechanical dispersion was similar in both groups. Circumferential strains, twisting/untwisting mechanics were preserved.

**Conclusion:**

Our results strongly support that the cardiac morphological remodeling observed in our AN patients was associated with normal ventricular regional myocardial functions. Only GLS was higher in AN patients, but its clinical significance remains to be demonstrated.

## Introduction

Anorexia nervosa (AN) is characterized by important weight loss, severe malnutrition and body composition abnormalities (1). Most cases are observed in females aged between 12 and 25 years (1). Cardiovascular complications in AN are frequent, occurring in up to 80% of patients, and some can be life-threatening (2). The most common abnormalities reported are sinus bradycardia, hypotension and electrocardiographic abnormalities (3). A left ventricular (LV) remodeling has also been observed, characterized by a diminished wall thickness due to the loss of cardiac muscle (2). Regarding global diastolic function, a specific LV filling profile is usually observed, with decreased A wave and increased E/A ratio, on account very likely to sinus bradycardia (2, 4). Systolic function is often unchanged, with normal LV stroke volume and ejection fraction (EF) (5). However, most of studies in AN patients referred to standard ultrasound methods, precluding any conclusion regarding their myocardial regional function.

Myocardial strain analyses from speckle tracking echocardiography (STE) have emerged as a reliable technique for studying myocardial mechanics (6). Although they enable detecting subtle alterations in systolic function when EF is still preserved (6), their relative load dependency makes the myocardial deformation indices unable to account for changes in afterload observed in AN patients. To overcome this limitation, global myocardial work (GMW), estimated from construction of LV pressure-strain loops (LV-PSL), has recently emerged as an interesting echocardiographic tool for assessing the myocardial function (7–9). LV-PSL are strongly correlated with myocardial glucose metabolism (7) and thus could be altered in AN patients with caloric deprivations.

Non-uniform contractions of LV myocardial walls due to a decline of synchronicity affect myocardial performance. In AN patients, previous studies showed structural myocardial abnormalities with possible presence of fibrosis (10–12). Since strong interactions between myocardial fibrosis and conduction disorders have been demonstrated (13–15), we questioned a potential increase of LV mechanical dispersion in this specific population. The LV mechanical dispersion can be assessed not only from the segmental analysis of timing of LV strains (8, 13, 16), but also from the myocardial work, by differentiating the myocardial constructive work (MCW) and the myocardial wasted work (MWW) to estimate myocardial work efficiency (MWE) (7, 17).

Another important factor influencing LV myocardial performance is LV twist. During systole, contraction of the cardiomyocytes induced not only LV longitudinal, but also circumferential and radial strains (18, 19). Moreover, due to the complex orientation of the myocardial fibers, LV twists consequently to the opposing rotations of the base and the apex. Twisting of the LV, which could be assessed non-invasively by STE, is essential to maintain EF and efficiency of systolic function (20). It plays also a crucial role during diastole, with elastic energy stored during systole abruptly released during untwisting, generating an intraventricular pressure gradients very early in diastole and allowing filling to proceed at low filling pressure (18–20). It has been well-demonstrated that LV twisting mechanics was altered under various pathological states (20, 21), but its evaluation has never been done in AN patients.

In this context, the objective of the study was to compare the cardiac function of AN patients with healthy controls using novel advances in STE. We hypothesized that (1) LV strains and LV GMW would be decreased in AN patients, (2) LV intraventricular mechanical dispersion would be increased, and (3) LV twisting mechanisms would be enhanced to preserve their systolic function.

## Methods

### Study Population

The study, prospective, included female patients with AN who had been diagnosed in a pediatric department of a university hospital in France between March 2019 and January 2020. All patients ranged from 10 to 18 years and fulfilled the DSM V criteria for AN (American Psychiatric Association) (22). The control group was composed of healthy adolescent girls with normal body mass and free from eating disorders. The BMI z score was calculated for all participants by specific formula (23). Patients and control subjects with chronic disease, congenital heart defect, positive family history of cardiac disease were excluded. Written informed consent was obtained from the study participants and their guardians. The Ile de France Ethics Committee approved the protocol for this study (18.12.05.66738 CAT 2).

### Anthropometrical and Body Composition Assessments

Body height and body mass were measured. BMI was calculated as body mass.body height^−2^ and body surface area (BSA) was calculated according to Boyd (24). Blood pressure (BP) and HR were measured using an automatic device (General electric, Dynamap PRO 300 V2, Boston). Bradycardia was defined by HR <50 bpm, as in guideline on the evaluation and management of patients with bradycardia (Task force of 2018) (25). Systolic arterial hypotension was defined by systolic BP (SBP) below the 5^th^ percentile and/or 90 mmHg (26). Body composition, including body fat mass with abdominal fat thickness (AFT) and lean mass, was evaluated by a bio-impedance system, validated for the measurement of body composition in children (27) (BioparHom, Z-Métrix, France).

### Echocardiographic Recordings

Echocardiography was carried out with the subject in left lateral decubitus position, with Vivid Q ultrasound systems (GE Healthcare, Horten, Norway) using a 3.5-MHz transducer (M4S probe). Cine loops were recorded in parasternal long axis and apical (5, 4, 3 and 2 chambers) views and saved for blinded offline analysis (EchoPac, BT203 version, GE Healthcare). Grayscale images were saved at a frame rate of 80–90 frames.sec^−1^ and color tissue velocity images at a frame rate of 120–140 frames.sec^−1^. 2D echocardiographic measurements were performed in accordance with the guidelines of the American Society of Echocardiography (28). All echocardiographic data were averaged from measurements obtained on 3–5 cardiac cycles.

### LV Morphology and Global Function

LV diameters and myocardial thickness were measured from the parasternal long axis view. LV mass was estimated using Devereux formula and indexed to height^2.7^ as recommended in the pediatric population (29, 30). LV diastolic function was assessed from peak early (E wave) and atrial (A wave) transmitral flow velocities and from peak E' and A' TDI velocities, at the mitral annular level in the different apical views. LV volumes and EF were assessed using the Simpson's biplane method. Stroke volume and cardiac output (CO) were assessed from 5 chamber and parasternal long axis views, and then indexed to the BSA. Systemic vascular resistances were calculated by the formula mean arterial pressures divided by cardiac output, according the Poiseuille Law.

### LV Global, Regional Strains and Dyssynchrony

Global longitudinal strain (GLS), circumferential strains (CS), diastolic longitudinal strain rate (LSr_diast_) and twist mechanics (apical and basal rotations, peak twist and untwisting rate) were obtained as previously detailed (31). Data were normalized to percentage of systolic duration to avoid differences in heart rate and cine loop frame rates and to provide averaged strain curves during the cardiac cycle for AN and control groups. LV twist was calculated as the difference between apical and basal rotations at each percentage of systolic duration and untwist, occurring during diastole, was calculated. In order to assess the dynamics of global LV twist and its relation to radial displacement throughout the cardiac cycle, we constructed twist-displacement loops. Averaged radial displacement data from six segments in basal and in apical short-axis planes were summed and divided by 2 to obtain the mean value of radial displacement. We considered GLS, circumferential strains and LV twist as indices of myocardial systolic function, LSr_diast_ as index of LV relaxation (32) and peak untwisting rate as an index of LV diastolic suction.

### LV Mechanical Dispersion and Myocardial Work Quantification

From a 17-segments model from apical 4, 3 and 2 chamber views to assess LV regional strains and time to peak (TTP) strains (18), LV mechanical dispersion was assessed by the standard deviation (SD) of the TTP (in ms) over the 17 segments (SD _17S_).

**Figure 1:**
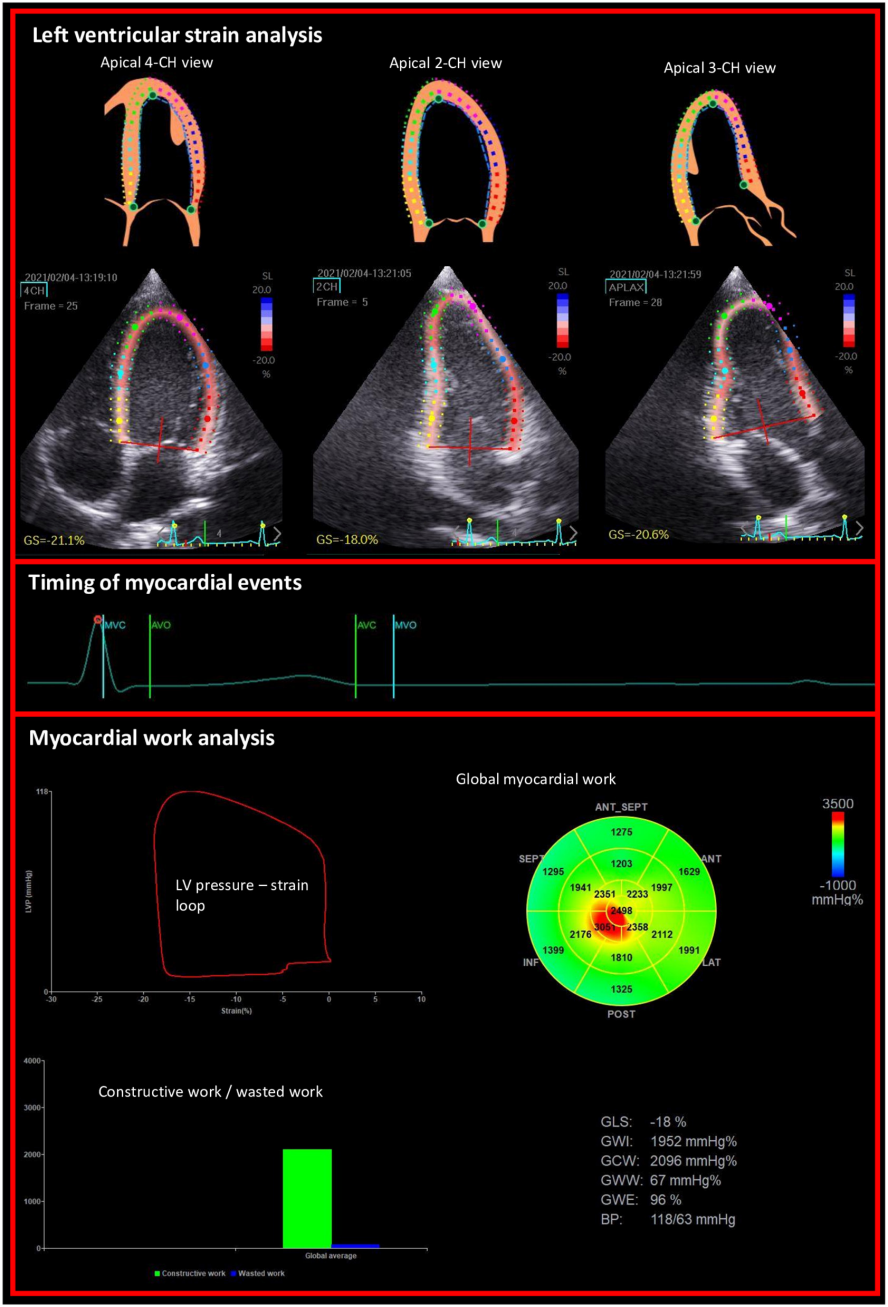
Example of non-invasive estimation of the LV myocardial work from the left ventricular longitudinal strains on apical views and the timing of mitral and aortic valve events.

Myocardial work (MW) and related parameters were estimated using the Automatic function imaging of the EchoPac software (Figure 1). MW was estimated as a function of time throughout the cardiac cycle by the combination of LV strain data (recorded on the apical 4, 3 and 2 chambers) obtained by STE and a non-invasively estimated LV pressure curve as described and validated by Russell et al. (7, 8). Peak arterial pressure measured with a cuff-manometer was assumed to be equal to peak systolic and diastolic LV pressures and to be uniform throughout the ventricle. MW was then quantified by calculating the rate of segmental shortening by differentiating the strain curve and multiplying the resulting value by the instantaneous LV pressure.

MW was calculated from mitral valve closure until mitral valve opening. During LV systole, segmental shortening contributes to the final LV ejection, whereas segmental stretch or lengthening does not contribute to LV ejection. As a result, the work performed by the myocardium during segmental shortening represents MCW, whereas the work performed by the myocardium during stretch or segmental lengthening represents energy loss, which is defined as MWW (9). During isovolumic relaxation, segmental lengthening contributes to LV relaxation, whereas segmental shortening does not. As a result, the work performed by the myocardium during segmental shortening, which does not promote LV relaxation, was considered MWW (9). On the opposite, the work performed by the myocardium during segmental lengthening was considered segmental MCW (9). The global MWE was finally obtained as follow:


$$\begin{array}{l} MWE\;=\frac{MCW}{MCW+MWW}\;\times \;100 \end{array}$$

### Statistical Analysis

All values were expressed as mean ± SD. Statistical analyses were performed using Medcalc (version 19.1, Medcalc Software). One-way analysis of variance (ANOVA) was used to compare groups, after checking the normality of distribution of each variable by Shapiro-Wilk test. In the absence of normal distribution, the nonparametric Kruskal-Wallis test was used. Statistical significance for all analyses was assumed at *p* < 0.05.

## Results

### Clinical Characteristics

Twenty-six female AN patients, with an illness duration of 19 ± 9 months, and 33 controls were included. Table 1 shows the main clinical characteristics and the body composition of AN patients and controls. As expected, patients with AN had significantly lower body mass, BMI and BSA than controls. Percentage of body fat mass was lower and of lean mass was higher in AN patients compared to controls. AN patients showed significantly lower resting HR and lower systolic BP than the controls. Eight AN patients (30.8 %) had sinus bradycardia and 7 (26.9 %) had systolic hypotension.

**Table 1:** Baseline characteristics of AN patients and controls.

	**AN patients (*n* = 26)**	**Controls (*n* = 33)**
Age (years)	14.6 ± 1.9	14.1 ± 2.0
Tanner's pubertal stage	3.4 ± 1.1	4.0 ± 1.3
**Anthropometry**		
Height (cm)	159.8 ± 9.1	162.6 ± 10.0
Body mass (kg)	40.7 ± 8.2^***^	51.2 ± 9.8
BMI (kg.m^−2^)	15.8 ± 2.1^***^	19.2 ± 2.3
BSA (m^2^)	1.33 ± 0.16^***^	1.52 ± 0.19
**Bioimpedance analysis**
AFT (mm)	8.4 ± 3.9^**^	14.1 ± 7.0
Body fat mass (kg)	5.1 ± 3.4^***^	10.5 ± 4.8
Body fat mass (%)	11.8 ± 6.2^***^	20.0 ± 6.6
Lean body mass (%)	85.0 ± 6.3^***^	76.6 ± 6.4
**Hemodynamic constants**		
Heart rate (bpm)	56 ± 12^***^	74 ± 11
Systolic BP (mmHg)	99 ± 14^***^	110 ± 8
Diastolic BP (mmHg)	63 ± 11	67 ± 7
Mean BP (mmHg)	75 ± 12^*^	81 ± 7

*AN, anorexia nervosa; BMI, body mass index; BSA, body surface area; AFT, abdominal fat thickness; BP, blood pressure; Values are mean ± SD. Significant differences with controls*

* *p < 0.05*;

** *p < 0.01*;

*** *p < 0.001*.

### Left Ventricular Morphology and Function

Data of LV morphology and function are presented in Table 2. LV posterior wall thickness, LV mass, LV mass index to height^2.7^ were lower in AN patients compared to controls. There was no difference in the LV septum thickness, relative wall thickness (RWT), LV end-diastolic and end-systolic volume. AN patients presented with lower CO index, lower A wave with higher E/A ratio. Stroke volume index and EF were not different between groups. Systemic vascular resistances were higher in AN patients compared to controls.

**Table 2:** Left ventricular morphology and function in AN patients and controls.

	**AN patients (*n* = 26)**	**Controls (*n* = 33)**
**Morphology**		
LV septum thickness (cm)	0.72 ± 0.15	0.77 ± 0.12
LV posterior wall thickness (cm)	0.67 ± 0.12^*^	0.75 ± 0.12
RWT	0.33 ± 0.06	0.36 ± 0.05
LV end-diastolic volume (mL)	79 ± 19	86 ± 18
LV end-systolic volume (mL)	29 ± 8	31 ± 7
LV mass (g)	79 ± 26^*^	96 ± 24
LV mass index to height ^2.7^ (g.m^−2.7^)	22 ± 6^*^	26 ± 5
**Function**		
**LV systolic function**		
Stroke volume index (mL.m^−2^)	36.9 ± 5.9	35.6 ± 5.1
CO index (L.min^−1^.m^−2^)	2.0 ± 0.4^***^	2.5 ± 0.4
EF (%)	64 ± 5	64 ± 6
**LV diastolic function**
E wave (cm.s^−1^)	84 ± 17	82 ± 14
A wave (cm.s^−1^)	30 ± 6^***^	41 ± 7
E/A	2.9 ± 0.9^***^	2.0 ± 0.5
E' (cm.s^−1^)	12.8 ± 1.4	13.5 ± 1.3
A' (cm.s^−1^)	4.2 ± 0.9^***^	5.6 ± 1.1
E'/A'	3.2 ± 0.7^***^	2.5 ± 0.5
**Systemic vascular resistances (AU)**	30.1 ± 8.2^***^	21.9 ± 5.4

*Values are mean ± SD. Significant differences with controls*

* *p < 0.051*;

^**^p < 0.01;

*** *p < 0.001*.

*AN, anorexia nervosa; LV, left ventricular; RWT, relative wall thickness; CO, cardiac output; EF, ejection fraction; AU, arbitrary unit*.

### Left Ventricular Strains and Myocardial Work

**Figure 2:**
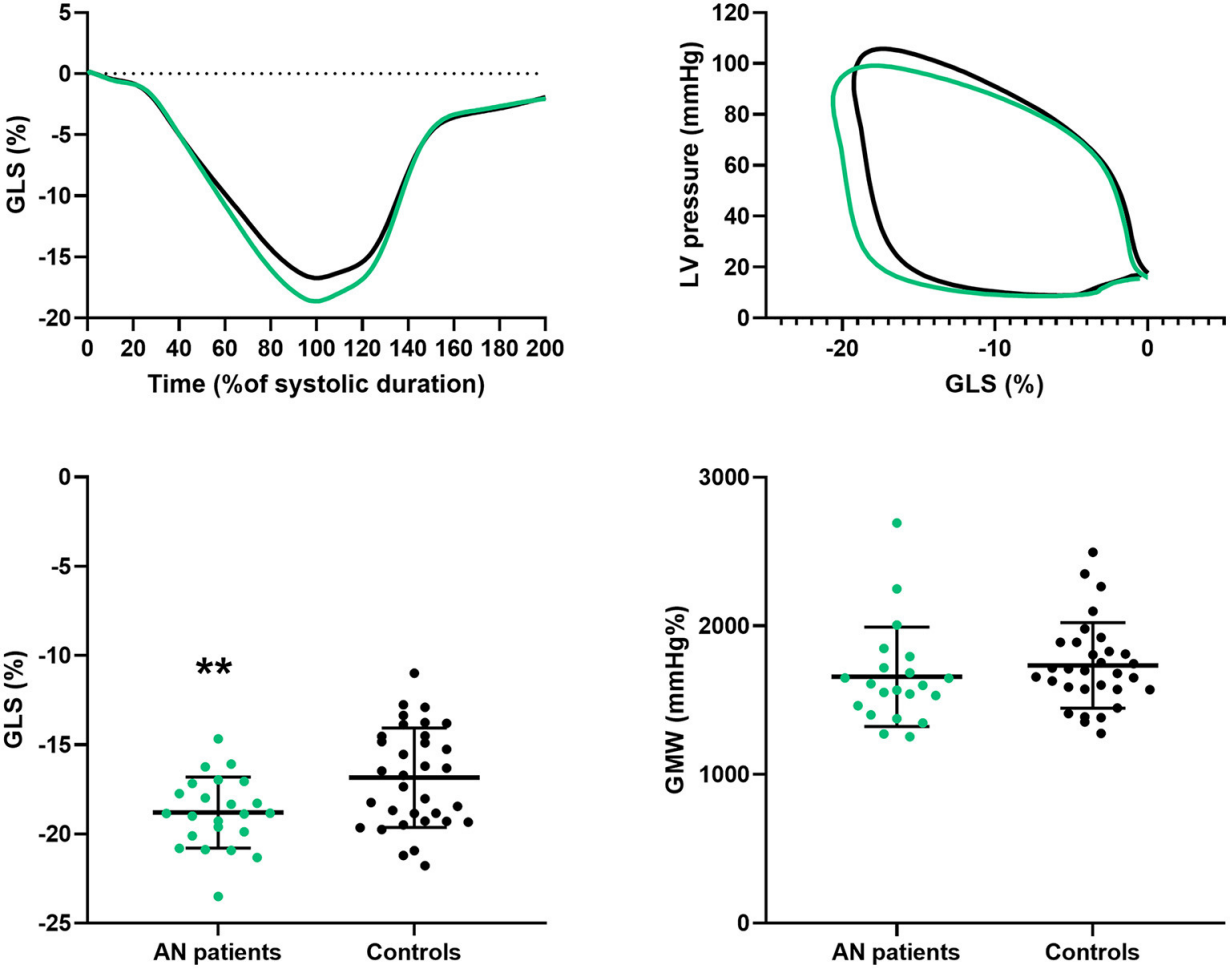
Longitudinal strain and global myocardial work in AN patients and controls. GLS, global longitudinal strain; LV, left ventricular; GMW, global myocardial work. Difference between groups: **P < 0.01.

LV strains and MW are presented in Figure 2 and Table 3. GLS was higher in AN patients compared to controls, whereas no difference were observed on circumferential strains. In AN patients, the LV-PSL was shifted to the left and downwards, consequently to the decrease of their LV pressures and the increase of their GLS. However, the area under the loop, reflecting the GMW, was similar between groups (1,657 ± 335 vs 1,737 ± 287 mmHg.% in AN patients and controls, respectively).

**Table 3:** Left ventricular strains and twisting mechanics in AN patients and controls.

	**AN patients (*n* = 26)**	**Controls (*n* = 33)**
**Systolic strain**		
GLS (%)	−18.8 ± 2.0^**^	−16.9 ± 2.8
**CS (%)**		
Basal level	−21.9 ± 2.9	−23.1 ± 3.6
Apical level	−29.6 ± 4.6	−30.9 ± 4.4
**Rotation (** **°** **)**		
Basal level	−3.3 ± 1.7	−3.7 ± 2.0
Apical level	5.8 ± 2.6	5.7 ± 2.1
Twist (°)	7.3 ± 3.2	7.5 ± 3.3
**Diastolic strain**		
LSr _diastolic_	1.9 ± 0.3	1.9 ± 0.5
Untwisting rate (°.s^−1^)	−67.5 ± 31.9	−80.3 ± 31.5

Values are mean ± SD. Significant differences with controls ^*^p < 0.05,

** *p < 0.01*,

^***^p < 0.001.

*AN, anorexia nervosa; GLS, global longitudinal strain; CS, circumferential strain; LSr, longitudinal strain rate*.

### Left Ventricular Mechanical Dispersion

The SD_17S_, an overall marker of intraventricular mechanical dispersion, was similar between groups (Table 4). No difference was observed between groups regarding MCW. The MWW was also similar and consequently the MWE was unchanged.

**Table 4:** LV dyssynchrony assessed by LV strains and myocardial work in AN patients and controls.

	**AN patients**	**Controls**
	**(*n* = 26)**	**(*n* = 33)**
**Myocardial work**		
MCW	1,842.3 ± 336.7	2,014.0 ± 299.4
MWW	131.0 ± 78.3	173.0 ± 152.5
MWE	92.8 ± 3.8	91.9 ± 4.6
**LV Dyssynchrony**		
SD _17S_ (ms)	42.3 ± 24.3	45.8 ± 27.0

Values are mean ± SD. Significant differences with controls *p < 0.05; **p < 0.01; ***p < 0.001.

*AN, anorexia nervosa; MCW, myocardial constructive work; MWW, myocardial wasted work; MWE, myocardial work efficiency; SD, standard deviation*.

### LV Twisting Mechanics

**Figure 3:**
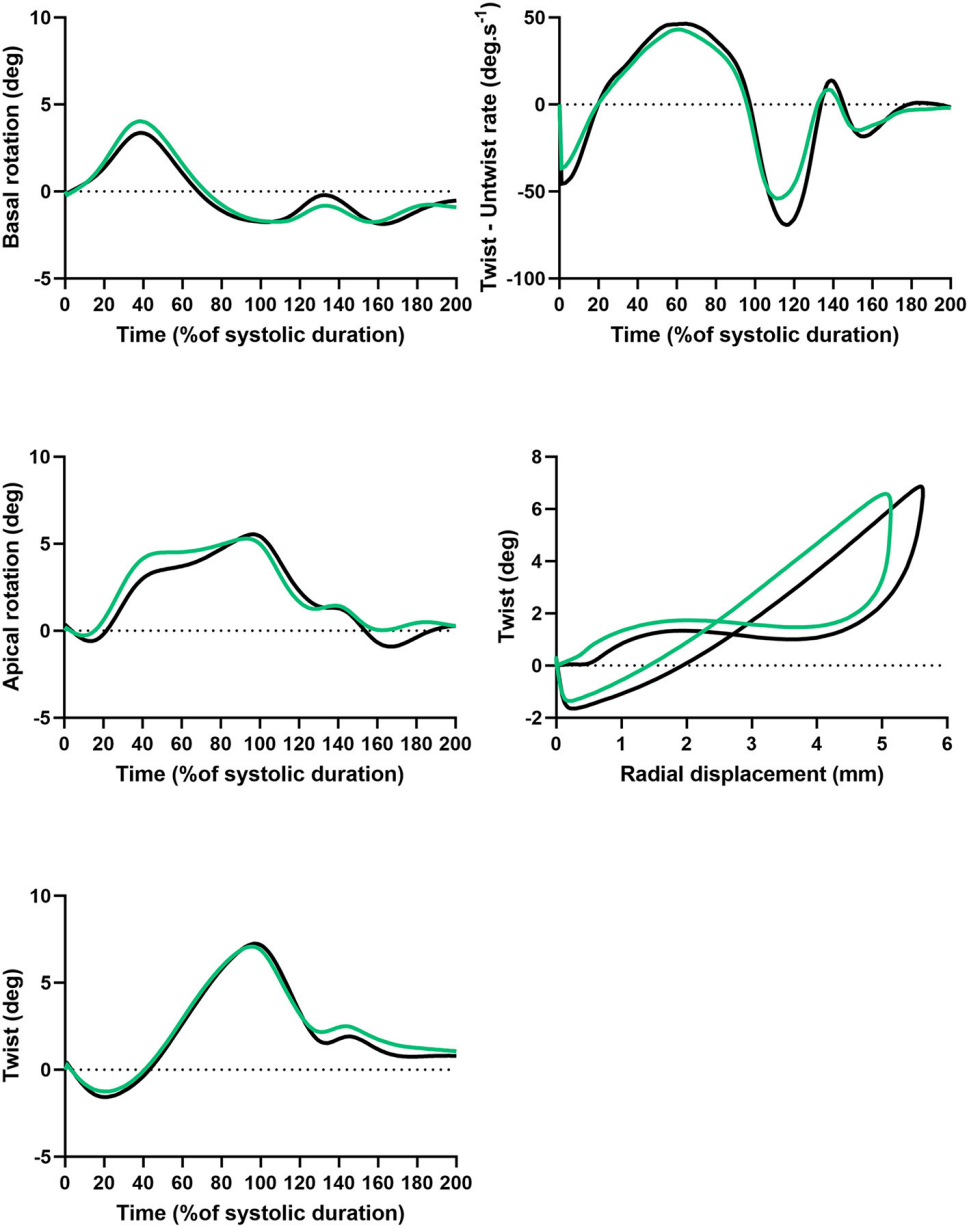
Twist and untwist mechanisms in AN patients and controls. Basal rotation, apical rotation and twist, function of time (%of systolic duration). Twist – untwist rate, function of time (% of systolic duration). Twist function of radial displacement.

LV twisting mechanics are presented in Figure 3 and Table 3. LV rotations and twist were similar between groups. Peak untwisting rate tended to be lower in AN patients despite difference did not reached statistical significance. Twist-radial displacement loops showed a similar pattern in both groups. After a small initial clockwise twist at the onset of ejection, twist increased linearly throughout systole. Early diastole was characterized by rapid untwisting despite small radial displacement, and then untwisting was smaller whereas displacement was larger from mid to late diastole. These results strongly supported that the untwisting efficiency and the suction effect was preserved in AN patients.

## Discussion

It was well-described that AN was associated with cardiac alterations (3, 5, 33). In our study, we confirmed that AN patients had lower LV mass and lower CO secondary to bradycardia. Using STE, the main objective of our study was to get insight into their regional myocardial function, including MW and LV twisting mechanics. We observed that, in AN patients, (1) GLS was higher but GMW unchanged, (2) intraventricular mechanical dispersion, assessed from time to peak strains and MWE, remained similar compared to controls, (3) twisting mechanics were preserved.

### Higher Global Longitudinal Strain but Unaltered Global Myocardial Work in AN Patients

The first main result of our study was that the GLS, which allows a more sensitive analysis of systolic function than EF (34), was significant higher in AN patients compared to controls. This is an unexpected result since in various pathological states the GLS is usually decreased (35). To our knowledge, only one previous study evaluated GLS in AN patients and found a similar GLS between AN patients and controls (36). Discrepancies from our results could be explained by a different BMI z score between our group and their two groups of AN patients (i.e., −1.78 vs. −0.44 and −1.01). The severity of the disease could have an impact on the LV myocardial function.

The increase in GLS could results from a complex interplay between several factors including hypotension, low resting HR and cardiac hypotrophy. In the present study, we assessed the MW, a new echocardiographic tool based on both an assessment of LV strain and an estimation of LV intraventricular pressures (9, 37). Contrary to GLS, we did not observed difference on GMW between our AN patients and controls. Indeed, despite the different pressure-strain loops (i.e., shifted to the left and downward in AN patients due to their higher strains and their lower SBP), area, representing the GMW, were similar between the 2 groups. This second main result strongly supported that the increase in GLS of AN patients were probably linked with their lower SBP. The key role of cardiac afterload on GLS has been demonstrated in hypertensive patients or in animals in which a decrease of GLS was observed consequently to an increase in afterload (35, 38, 39). Low HR in our AN patients could also be involved in their higher GLS, as observed in endurance athletes, in whom resting bradycardia was associated with an increase in GLS (35, 38). In pediatric populations, advancing age leads to a decrease of HR concomitantly with an increase of GLS (40), suggesting also a potential link between HR and GLS. The low LV mass of our AN patients could be another factor explaining their increased GLS. Indeed, in animals, the increase in the LV dimensions was associated with an decrease in GLS (39). In our study, LV mass of patients with AN was lower compared to controls. A previous study observed that the lower LV mass in patient with AN was linked to high growth hormon, low IGF-1 and low thyroid hormon levels (41). However, our results failed to demonstrate significant correlations between GLS and posterior wall thickness, LV mass or SBP.

The clinical significance of the increased GLS remains unclear. Despite this is purely speculative, the increase in GLS at rest could act to favor the stroke volume and thus the CO despite their low LVM and resting bradycardia. We could also question their reserve of GLS during exercise and thus their effort tolerance, since it has been well-described that in response to physical activity (42, 43) or under dobutamine stress (44) the increase in LV strains acted to maintain or improved the stroke volume (42). In this context, further studies would be helpful to understand how the specific heart of AN patients respond to exercise.

### Intraventricular Mechanical Dispersion in AN Patients

Several case studies based on autopsy reported cardiac fibrosis in AN patients (10, 11). Interestingly, using cardiac MRI (12) and late gadolinium enhancement, Oflaz et al. (12) observed that 23% of AN patients had a presence of myocardial fibrosis (12). Since fibrosis is known to favor conduction abnormalities (13–15), we questioned if our AN patients had a higher LV mechanical dispersion compared to controls. Based on segmental STE analysis, we observed that the SD_17S_, an overall index of LV mechanical dispersion remained unchanged in AN patients. Additionally, we used an up-to-date method by quantifying both MCW and MWW to calculate MWE (8, 13). In patients with increased mechanical dispersion, MWW, work that is being done by the ventricle but does not contribute to LV ejection (8, 9), is significantly increased and MWE reduced (8, 9, 13). In our study, we did not find an increase in MWW in AN patients compared to controls, highlighting that their intraventricular mechanical dispersion was not increased.

### Left Ventricular Twisting Mechanics in AN Patients

During systole, contraction of the cardiomyocytes generates opposite rotations of the LV base and the apex, inducing a LV twist (18–20). Despite its key role in both systolic and diastolic performance, twist mechanics have been largely neglected in previous studies. We hypothesized that LV rotations and twist could be modified in AN patients, since it was well-demonstrated that LV twist can be affected by changes in loading conditions (21, 45). More specifically, LV twist decreased when afterload increased and/or preload decreased (21). Another major finding of our study was that LV rotations and twist was unaltered in AN patients. This could be an additional mechanism favoring the maintenance of EF and stroke volume since LV twist, helps for bringing a uniform distribution of LV fiber stress and fiber shortening across the wall, and its disappearance has been clearly shown to increase oxygen demand and reduce the efficiency of LV systolic function (20, 21).

The LV twist allows energy to be stored in elastic component during systole, energy restored very early in diastole, creating an intraventricular pressure gradient that favors LV filling (45, 46). The LV twist-untwist thus links systole to diastole (i.e., systolic-diastolic coupling). In our AN patients, LV untwisting was preserved. The twist-displacement loops highlighted that, in both groups, substantial untwisting occurred despite a relatively small radial displacement in early diastole, whereas untwisting was markedly smaller during the late phases of diastole in spite of substantial radial displacement. This result strongly supported that, in AN patients, the ventricle untwists, rapidly recoiling, creating a diastolic suction that was probably fully effective, contributing to their normal LV diastolic function.

## Conclusion

In our study, AN was accompanied by reduced HR and SBP, associated with cardiac remodeling characterized by a decrease in LV mass and wall thickness. However, the assessment of LV strains and MW, and also of LV twisting mechanics, brought new evidences that the cardiac function of adolescent AN patients was preserved. Of note, our study population was characterized by low BMI z score, but with a relatively short illness duration. So, it would be interesting to evaluate in the future the myocardial function of AN patients with a longer illness duration.

## Data Availability Statement

The original contributions presented in the study are included in the article/supplementary material, further inquiries can be directed to the corresponding author/s.

## Ethics Statement

The studies involving human participants were reviewed and approved by Ile de France Ethics Comittee (18.12.05.66738 CAT 2). Written informed consent to participate in this study was provided by the participants' legal guardian/next of kin.

## Author Contributions

JP: design, methodology, investigation, analysis, and writing initial manuscript. EM: investigation and supervision. DT: methodology and investigation. AC: analysis and supervision. ER: supervision. PO: methodology and analysis. SN: design, methodology, investigation, analysis, writing, and supervision. All authors contributed to the article and approved the submitted version.

## Funding

This work was supported by the Platform 3A, funded by the European Regional Development Fund, the French Ministry of Research, Higher Education and Innovation, the Provence-Alpes-Côte-d'Azur region, the Departmental Council of Vaucluse and the Urban Community of Avignon.

## Conflict of Interest

The authors declare that the research was conducted in the absence of any commercial or financial relationships that could be construed as a potential conflict of interest.

## Publisher's Note

All claims expressed in this article are solely those of the authors and do not necessarily represent those of their affiliated organizations, or those of the publisher, the editors and the reviewers. Any product that may be evaluated in this article, or claim that may be made by its manufacturer, is not guaranteed or endorsed by the publisher.

## References

[1] Giovinazzo S Sukkar SG Rosa GM Zappi A Bezante GP Balbi M. Anorexia nervosa and heart disease: a systematic review. Eat Weight Disord EWD. (2019) 24:199–207. 10.1007/s40519-018-0567-1 30173377

[2] Escudero CA Potts JE Lam PY De Souza AM Mugford GJ Sandor GGS. An echocardiographic study of left ventricular size and cardiac function in adolescent females with anorexia nervosa: LV size and cardiac function in AN. Eur Eat Disord Rev. (2016) 24:26–33. 10.1002/erv.2409 26449643

[3] Sachs KV Harnke B Mehler PS Krantz MJ. Cardiovascular complications of anorexia nervosa: a systematic review. Int J Eat Disord. (2016) 49:238–48. 10.1002/eat.22481 26710932

[4] Galetta F Franzoni F Cupisti A Morelli E Santoro G Pentimone F. Early detection of cardiac dysfunction in patients with anorexia nervosa by tissue Doppler imaging. Int J Cardiol. (2005) 101:33–7. 10.1016/j.ijcard.2004.03.006 15860380

[5] Olivares JL Vázquez M Fleta J Moreno LA Pérez-González JM Bueno M. Cardiac findings in adolescents with anorexia nervosa at diagnosis and after weight restoration. Eur J Pediatr. (2005) 164:383–6. 10.1007/s00431-005-1647-6 15909184

[6] Potter E Marwick TH. Assessment of left ventricular function by echocardiography: the case for routinely adding global longitudinal strain to ejection fraction. JACC Cardiovasc Imaging. (2018) 11:260–74. 10.1016/j.jcmg.2017.11.017 29413646

[7] Russell K Eriksen M Aaberge L Wilhelmsen N Skulstad H Remme EW. A novel clinical method for quantification of regional left ventricular pressure–strain loop area: a non-invasive index of myocardial work. Eur Heart J. (2012) 33:724–33. 10.1093/eurheartj/ehs016 22315346 PMC3303715

[8] Russell K Eriksen M Aaberge L Wilhelmsen N Skulstad H Gjesdal O. Assessment of wasted myocardial work: a novel method to quantify energy loss due to uncoordinated left ventricular contractions. Am J Physiol-Heart Circ Physiol. (2013) 305:H996–H1003. 10.1152/ajpheart.00191.2013 23893165

[9] Samset E Healthcare G. Evaluation of Segmental Myocardial Work in the Left Ventricle.4. Available online at: https://www.gehealthcare.com/-/media/8cab29682ace4ed7841505f813001e33.pdf

[10] Lamzabi I Syed S Reddy VB Jain R Harbhajanka A Arunkumar P. Myocardial changes in a patient with anorexia nervosa. Am J Clin Pathol. (2015) 143:734–7. 10.1309/AJCP4PLFF1TTKENT 25873509

[11] SHORT PAPERS Anorexia Nervosa and Sudden Death.

[12] Oflaz S Yucel B Oz F Sahin D Ozturk N Yaci O. Assessment of myocardial damage by cardiac MRI in patients with anorexia nervosa. Int J Eat Disord. (2013) 46:862–6. 10.1002/eat.22170 23922168

[13] Nguyên UC Verzaal NJ van Nieuwenhoven FA Vernooy K Prinzen FW. Pathobiology of cardiac dyssynchrony and resynchronization therapy. EP Eur. (2018) 20:1898–909. 10.1093/europace/euy035 29771311

[14] Anderson KP Walker R Urie P Ershler PR Lux RL Karwandee SV. Myocardial electrical propagation in patients with idiopathic dilated cardiomyopathy. J Clin Invest. (1993) 92:122–40. 10.1172/JCI116540 8325977 PMC293548

[15] Kawara T Derksen R de Groot JR Coronel R Tasseron S Linnenbank AC. Activation Delay After Premature Stimulation in Chronically Diseased Human Myocardium Relates to the Architecture of Interstitial Fibrosis. Circulation. (2001) 104:3069–75. 10.1161/hc5001.100833 11748102

[16] Cvijic M Duchenne J Ünlü S Michalski B Aarones M Winter S. Timing of myocardial shortening determines left ventricular regional myocardial work and regional remodelling in hearts with conduction delays. Eur Heart J - Cardiovasc Imaging. (2018) 19:941–9. 10.1093/ehjci/jex325 29272366

[17] Schrub F Schnell F Donal E Galli E. Myocardial work is a predictor of exercise tolerance in patients with dilated cardiomyopathy and left ventricular dyssynchrony. Int J Cardiovasc Imaging. (2019) 36:45–53. 10.1007/s10554-019-01689-4 31515694

[18] Thomas JD Popović ZB. Assessment of left ventricular function by cardiac ultrasound. J Am Coll Cardiol. (2006) 48:2012–25. 10.1016/j.jacc.2006.06.071 17112991

[19] Sengupta PP Korinek J Belohlavek M Narula J Vannan MA Jahangir A. Left ventricular structure and function: basic science for cardiac imaging. J Am Coll Cardiol. (2006) 48:1988–2001. 10.1016/j.jacc.2006.08.030 17112989

[20] Bloechlinger S Grander W Bryner J Dünser MW. Left ventricular rotation: a neglected aspect of the cardiac cycle. Intensive Care Med. (2011) 37:156–63. 10.1007/s00134-010-2053-8 20878386

[21] Stöhr EJ Shave RE Baggish AL Weiner RB. Left ventricular twist mechanics in the context of normal physiology and cardiovascular disease: a review of studies using speckle tracking echocardiography. Am J Physiol Heart Circ Physiol. (2016) 311:H633–644. 10.1152/ajpheart.00104.2016 27402663

[22] Loas G. The DSM-V : an overview. Rev Med Brux. (2016) 37:231–4. 28525220

[23] Rolland-Cachera MF Cole TJ Sempé M Tichet J Rossignol C Charraud A. Body Mass Index variations: centiles from birth to 87 years. Eur J Clin Nutr. (1991) 45:13–21. 1855495

[24] Orimadegun A Omisanjo A. Evaluation of five formulae for estimating body surface area of nigerian children. Ann Med Health Sci Res. (2014) 4:889–98. 10.4103/2141-9248.144907 25506482 PMC4250987

[25] Kusumoto FM Schoenfeld MH Barrett C Edgerton JR Ellenbogen KA Gold MR. 2018 ACC/AHA/HRS Guideline on the Evaluation and Management of Patients With Bradycardia and Cardiac Conduction Delay: A Report of the American College of Cardiology/American Heart Association Task Force on Clinical Practice Guidelines and the Heart Rhythm Society. Circulation. (2019) 140:e382–e482. 10.1161/CIR.0000000000000628 30586772

[26] Banker A Bell C Gupta-Malhotra M Samuels J. Blood pressure percentile charts to identify high or low blood pressure in children. BMC Pediatr. (2016) 16:98. 10.1186/s12887-016-0633-7 27430884 PMC4950817

[27] Barreira TV Staiano AE Katzmarzyk PT. Validity assessment of a portable bioimpedance scale to estimate body fat percentage in white and African-American children and adolescents. Pediatr Obes. (2013) 8:e29–32. 10.1111/j.2047-6310.2012.00122.x 23239610 PMC3602331

[28] Lang RM Badano LP Mor-Avi V Afilalo J Armstrong A Ernande L. Recommendations for cardiac chamber quantification by echocardiography in adults: an update from the American Society of Echocardiography and the European Association of Cardiovascular Imaging. Eur Heart J – Cardiovasc Imaging. (2015) 16:233–71. 10.1093/ehjci/jev014 25712077

[29] de Simone G Daniels SR Devereux RB Meyer RA Roman MJ de Divitiis O. Left ventricular mass and body size in normotensive children and adults: assessment of allometric relations and impact of overweight. J Am Coll Cardiol. (1992) 20:1251–60. 10.1016/0735-1097(92)90385-Z 1401629

[30] Devereux RB Alonso DR Lutas EM Gottlieb GJ Campo E Sachs I. Echocardiographic assessment of left ventricular hypertrophy: comparison to necropsy findings. Am J Cardiol. (1986) 57:450–8. 10.1016/0002-9149(86)90771-X 2936235

[31] Maufrais C Schuster I Doucende G Vitiello D Rupp T Dauzat M. Endurance training minimizes age-related changes of left ventricular twist-untwist mechanics. J Am Soc Echocardiogr. (2014) 27:1208–15. 10.1016/j.echo.2014.07.007 25127983

[32] Wang J Khoury DS Thohan V Torre-Amione G Nagueh SF. Global diastolic strain rate for the assessment of left ventricular relaxation and filling pressures. Circulation. (2007) 115:1376–83. 10.1161/CIRCULATIONAHA.106.662882 17339549

[33] Gottdiener JS Gross HA Henry WL Borer JS Ebert MH. Effects of self-induced starvation on cardiac size and function in anorexia nervosa. Circulation. (1978) 58:425–33. 10.1161/01.CIR.58.3.425 679432

[34] Al Saikhan L Park C Hardy R Hughes A. Prognostic implications of left ventricular strain by speckle-tracking echocardiography in the general population: a meta-analysis. Vasc Health Risk Manag. (2019) 15:229–51. 10.2147/VHRM.S206747 31413582 PMC6661977

[35] A Test in Context: Myocardial Strain Measured by Speckle-Tracking Echocardiography. Elsevier Enhanced Reader. 10.1016/j.jacc.2016.12.012 28231932

[36] Morris R Prasad A Asaro J Guzman M Sanders L Hauck A. Markers of Cardiovascular Dysfunction in Adolescents With Anorexia Nervosa. Glob Pediatr Health. (2017) 4. 10.1177/2333794X17727423 28890913 PMC5580842

[37] Smiseth OA Donal E Penicka M Sletten OJ. How to measure left ventricular myocardial work by pressure-strain loops. Eur Heart J Cardiovasc Imaging. (2020) 22:259–261. 10.1093/ehjci/jeaa301 33257982

[38] Iudice FL Petitto M Ferrone M Esposito R Vaccaro A Buonauro A. Determinants of Myocardial Mechanics in Top-Level Endurance Athletes: Three-Dimensional Speckle Tracking Evaluation.7. 10.1093/ehjci/jew122 27325809

[39] Rösner A Bijnens B Hansen M How OJ Aarsaether E Müller S. Left ventricular size determines tissue Doppler-derived longitudinal strain and strain rate. Eur J Echocardiogr. (2009) 10:271–7. 10.1093/ejechocard/jen230 18827033

[40] Boettler P Hartmann M Watzl K Maroula E Schultemoenting J Knirsch W. Heart rate effects on strain and strain rate in healthy children. J Am Soc Echocardiogr. (2005) 18:1121–30. 10.1016/j.echo.2005.08.014 16275519

[41] Carlomagno G Mercurio V Ruvolo A Senatore I Halinskaya I Fazio V. Endocrine alterations are the main determinants of cardiac remodelling in restrictive anorexia nervosa. ISRN Endocrinol. (2011) 2011:171460. 10.5402/2011/171460 22363867 PMC3262625

[42] Izem O Maufrais C Obert P Rupp T Schuster I Nottin S. Kinetics of left ventricular mechanics during transition from rest to exercise. Med Sci Sports Exerc. (2019) 51:1838–44. 10.1249/MSS.0000000000002005 30973478

[43] Magne J Mahjoub H Dulgheru R Pibarot P Pierard LA Lancellotti P. Left ventricular contractile reserve in asymptomatic primary mitral regurgitation. Eur Heart J. (2014) 35:1608–16. 10.1093/eurheartj/eht345 24014387

[44] De Luca A Stolfo D Caiffa T Korcova R Barbati G Vitrella G. Prognostic Value of global longitudinal strain-based left ventricular contractile reserve in candidates for percutaneous correction of functional mitral regurgitation: implications for patient selection. J Am Soc Echocardiogr. (2019) 32:1436–43. 10.1016/j.echo.2019.07.006 31551186

[45] Sengupta PP Tajik AJ Chandrasekaran K Khandheria BK. Twist mechanics of the left ventricle: principles and application. JACC Cardiovasc Imaging. (2008) 1:366–76. 10.1016/j.jcmg.2008.02.006 19356451

[46] Notomi Y Popovic ZB Yamada H Wallick DW Martin MG Oryszak SJ. Ventricular untwisting: a temporal link between left ventricular relaxation and suction. Am J Physiol Heart Circ Physiol. (2008) 294:H505–513. 10.1152/ajpheart.00975.2007 18032523

