# Corrigendum: Left Ventricular Strains and Myocardial Work in Adolescents With Anorexia Nervosa

**DOI:** 10.3389/fcvm.2022.895870

**Published:** 2022-04-25

**Authors:** Justine Paysal, Etienne Merlin, Daniel Terral, Aurélie Chalard, Emmanuelle Rochette, Philippe Obert, Stéphane Nottin

**Affiliations:** ^1^Avignon University, LAPEC EA4278, Avignon, France; ^2^CHU Clermont-Ferrand, Néonatologie et réanimation pédiatrique, Clermont-Ferrand, France; ^3^CHU Clermont-Ferrand, Pédiatrie, Clermont-Ferrand, France; ^4^Université Clermont Auvergne, INSERM, CIC 1405, Unité CRECHE, Clermont-Ferrand, France; ^5^CHU Clermont-Ferrand, Cardiologie, Clermont-Ferrand, France

**Keywords:** myocardial strain, myocardial work, left ventricular mechanical dispersion, anorexia nervosa, left ventricular twist

The order of first and last names has been changed. The incorrect order was “Paysal Justine, Merlin Etienne, Terral Daniel, Chalard Aurélie, Rochette Emmanuelle, Obert Philippe and Nottin Stéphane.”

The correct order is “Justine Paysal, Etienne Merlin, Daniel Terral, Aurélie Chalard, Emmanuelle Rochette, Philippe Obert and Stéphane Nottin.”

The authors apologize for this error and state that this does not change the scientific conclusions of the article in any way. The original article has been updated.

## Publisher's Note

All claims expressed in this article are solely those of the authors and do not necessarily represent those of their affiliated organizations, or those of the publisher, the editors and the reviewers. Any product that may be evaluated in this article, or claim that may be made by its manufacturer, is not guaranteed or endorsed by the publisher.

